# Supplementary material for: Cycling reduces the entropy of neuronal activity in the human adult cortex
Source: PLoS One. 2024 Oct 2;19(10):e0298703. doi: 10.1371/journal.pone.0298703 (PMC11446439; doi:10.1371/journal.pone.0298703)
Supplement: Attachment — Submitted filename: Review_PLOS_CyclingEntropy.docx [file pone.0298703.s001.docx]

Review for “Cycling reduces the entropy of neuronal activity in the human adult cortex”

I would like to appreciate the unique idea behind this paper. There are certain amendments which I would want the authors to consider while revisiting this manuscript:

1. The abstract attempts to covers all the aspects of the topic, which is good. However, I do think that the first sentence which introduces EEG and the second sentence which instantly starts talking about the present study and recurrence entropy, without really making the connection.
2. When talking about Regions of Interest, the authors need to mention brain when saying ‘right and left sides’.
3. There is no connection created between entropy and cycling and the logic of connecting cycling with EEG or the very idea of doing this research. Why do we want to know about entropy? How is it beneficial?
4. The introduction is not impressive, as the authors directly begin with something that does not directly correlate academically with the title. The starting statement says “Richard Caton once said that the brain speaks for itself”, which is not impressive for a research paper.
5. Starting with the history of EEG is not a good idea, in the introduction. I would recommend to start with your main topic and why is it needed.
6. The comparison of walking with cycling holds interesting dynamics, however, it seems slightly inconclusive at the end on page 8.
7. From cycling, there is a sudden jump to gait without really defining and linking it with cycling.
8. The introduction section misses numerous links between ideas. How entropy impacts human beings, why would brain behave differently? why do we need to assess it, where can it be beneficial, why are we exploring it in cycling, why is there a need to do this?
9. Details of Sample is needed. What was the criteria for selecting these 13 women and 11 men?
10. The authors used the horizontal bicycle for the experiment. There is a need for citation here to justify the use of this bicycle in particular.
11. There is a need for a visual in the Methodology section which could exemplify the experimental paradigm and trials.
12. The logic, citation and justification for using eyes close and open is missing in the methodology section.
13. In the discussion section, the authors suddenly move from cycling to walking and its neuronal dynamics, which seems haphazard and requires to be connected well.
14. In the conclusion section, the topic has been linked with Parkinson’s disease at least 4 to 5 times. However, the sample taken for this study did not include this disease and its concerning manifestations. This makes the conclusion confusing for the reader to navigate. The authors need to revise this section with particular attention.
15. The statistical analysis requires more justification and details. Presently, it is very surficial and does not cover many reasons of how and why it was performed.
16. Many of the references are not according to the format. They need to be rechecked.
